# Standardization and optimization of force platform testing for balance assessment in cervical spondylotic myelopathy

**DOI:** 10.3389/fnins.2026.1905226

**Published:** 2026-09-10

**Authors:** Zheng Tian, Kaige Mao, Bin Zhang, Pengfei Chi, Junyao Cheng, Yifan Wang, Zhaohan Wang, Taoxu Yan, Bo Li, Jianheng Liu, Kai Song, Zheng Wang

**Affiliations:** 1 Department of Orthopedics, The Fourth Medical Center of Chinese PLA General Hospital, Beijing, China; 2 Medical School of Chinese PLA, Beijing, China

**Keywords:** balance assessment, center of pressure, center of pressure velocity, cervical spondylotic myelopathy, force platform

## Abstract

**Objective:**

This study aimed to compare the discriminative performance of different force platform testing methods for distinguishing patients with cervical spondylotic myelopathy (CSM) from asymptomatic controls and to evaluate the effects of arm position and test duration on individual balance parameters.

**Methods:**

In this cross sectional study, 56 patients with CSM and 50 asymptomatic volunteers were enrolled between March to December 2025. Balance function was measured with the eMat 500 force platform under two standing postures, arms hanging down and arms extended forward, both performed with the feet together and the eyes closed, and each posture was tested at durations of 15, 30, and 60s. Receiver operating characteristic (ROC) curve analysis was used to compare the diagnostic value of the different posture and duration combinations.

**Results:**

The diagnostic efficiencies of the six test protocols were all different for the four balance parameters. The center of pressure (COP) trajectory length and the mediolateral centre of gravity velocity performed best at 15 s with arms hanging down, the anteroposterior centre of gravity velocity performed best at 60s with arms extended forward, and the area of the 95% confidence ellipse performed best at 30s with arms extended forward. Combined ROC analysis ranked the overall diagnostic performance of the six protocols from best to worst as follows: 30s with arms hanging down (AUC = 0.897), 15 s with arms hanging down (AUC = 0.890), 30s with arms extended forward (AUC = 0.878), 60s with arms extended forward (AUC = 0.869), 60s with arms hanging down (AUC = 0.855), and 15 s with arms extended forward (AUC = 0.840).

**Conclusion:**

The research results indicate that the 30s both arms hanging down test has the highest diagnostic value. This testing approach has the potential to improve the efficiency of outpatient assessment for patients with CSM.

## 1 Introduction

Cervical spondylotic myelopathy (CSM) commonly causes compression and ischemia of the spinal cord, leading to injury of the corticospinal tract and subsequently gait abnormalities and balance dysfunction (Fouad et al., 2021; Matz, 2006; Rao et al., 2007). However, in the early stages of the disease, minor balance disorders is often ignored by patients and difficult to detect in a routine physical examination (Theodore, 2020; Mkorombindo et al., 2022). Studies have shown that force platform tests can accurately identify minor balance disorders in CSM patients. The use of this technology has slowly been introduced and implemented in the initial diagnosis and treatment of CSM patients. (Mkorombindo et al., 2022; Chen et al., 2021; Zhang et al., 2025; Kesler et al., 2024; Ver et al., 2020; Bergamin et al., 2014; Siasios et al., 2017; Maribo et al., 2012; Yoshikawa et al., 2008) At present, no standardized testing guidance have been established that should be followed when using force platforms to assess balance functions of CSM patients (Yoshikawa et al., 2008; Yano et al., 2020; Yano et al., 2022; Hassanzadeh et al., 2022; Haddas et al., 2021; Hasegawa and Dubousset, 2022). Most studies include a 30s test with feet together, eyes closed and arms extended forward (Mkorombindo et al., 2022; Kesler et al., 2024; Ver et al., 2020), others have allowed the arms to hang freely and have not limited the test to 30s (Mkorombindo et al., 2022). The absence of uniformity restricts the clinical applicability of force platform tests for CSM.

Accordingly, the present study was designed to evaluate the discriminative value of different force platform testing methods in CSM patients. We first compared the overall performance of each testing method in distinguishing CSM patients from asymptomatic controls by integrating four balance parameters. We then examined each balance parameter separately to determine whether its discriminative ability varied across testing method. Through these analyses, we aimed to identify a practical and sensitive testing method and to provide evidence to support the standardization of force platform based balance assessment in CSM testing methods.

## 2 Methods

### 2.1 General data

#### 2.1.1 Study subjects

A total of 219 people were recruited by our hospital from March to December 2025, consisting of 115 patients with CSM and 104 asymptomatic control group. Based on the preset exclusion criteria, 59 people were excluded from the CSM group and 54 from the control group; thus, a final sample of 56 patients with CSM and 50 healthy controls was obtained. CSM patients were selected based on the clinical diagnosis that met the criteria for symptoms, signs and imaging examination.

The exclusion criteria of CSM are as follows: (1) the presence of any disease other than CSM that could affect the function of balance, such as cerebrovascular stroke, parkinsonism or vestibular disorders; (2) past history of musculoskeletal injury or surgery; (3) alcoholism; (4) inability or unwillingness to complete the required tests.

The inclusion criteria of the asymptomatic control group: asymptomatic controls that were evaluated as not having CSM by professionals that are doctors. The exclusion criteria were identical to those of the CSM group.

This study was approved by the Ethics Committee of the Chinese PLA General Hospital and was conducted in accordance with local legislation and institutional requirements.

#### 2.1.2 Experimental equipment

The research involved the use of the eMat 500 force platform (model: FM500U HR 300010). Sensitivity of the sensor of the platform is 4 sensors/cm 2, the weight of the platform is 2.2 kg, maximum loading capacity is 200 kg, minimum loading capacity is 10 kg, it has a thickness of 5 mm and dimensions of 640 × 520 × 5 mm and 10,000 sensing points. The measurement platform is connected to the computer via the USB port and the FMap Analysis System v1.2.18.63 software is used to record and measure the indicators of balance parameters. The platform may also transform the balance behavior of the tester into numerical data.

### 2.2 Study methods

#### 2.2.1 Force platform balance testing procedures

**Figure 1:**
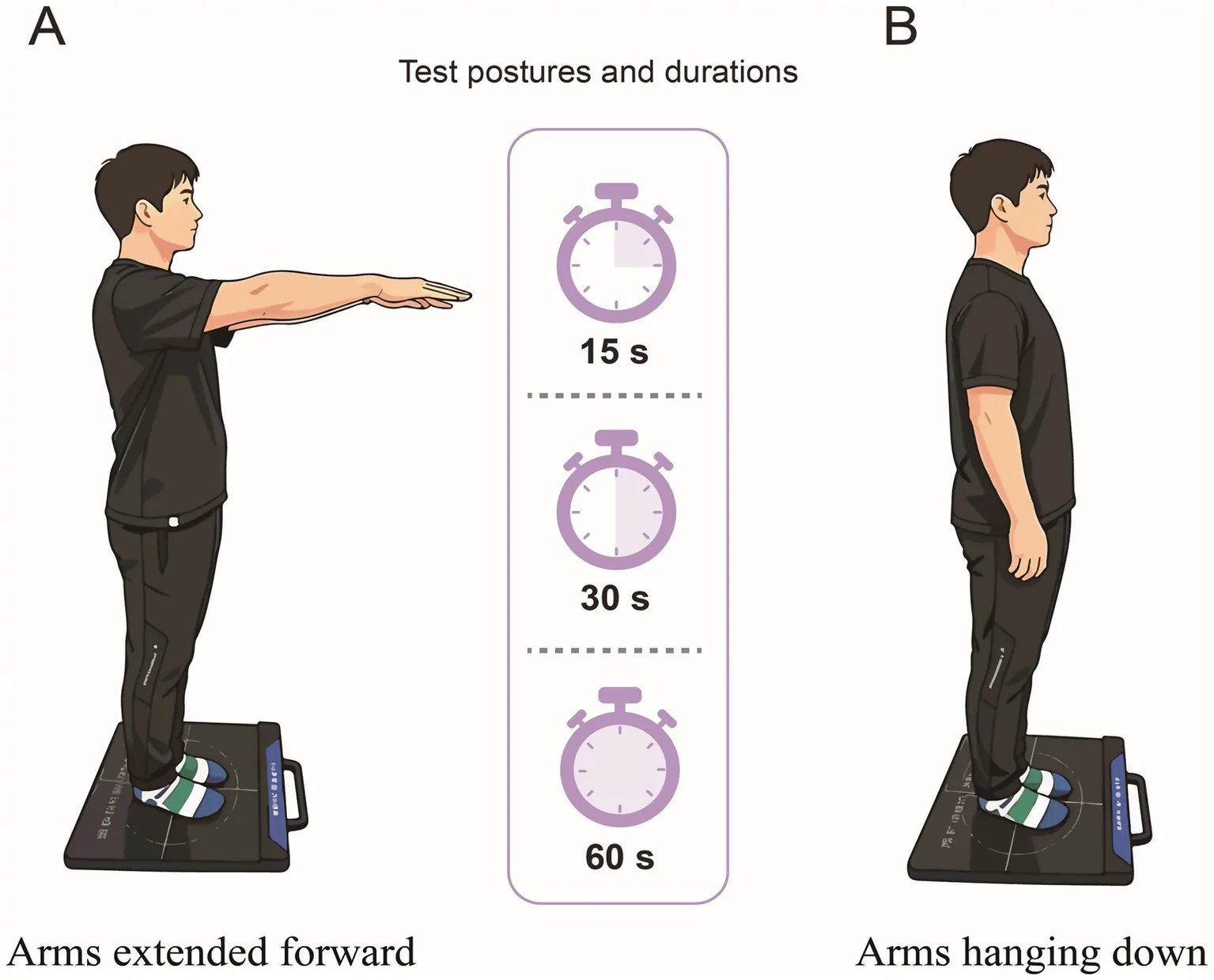
Force platform balance test: forward extended **(A)** and hanging down **(B)** arm postures.

Before testing, participants rested in a seated position for 5 min to minimize the influence of fatigue. The testing procedure and required postures were explained and demonstrated in a standardized manner. Before each assessment, the force platform was checked for proper operation according to the manufacturer’s instructions. Testing was performed in a quiet, well-lit room at a comfortable ambient temperature. Participants stood barefoot at the designated position on the platform with their feet together and eyes closed. For the arms hanging down condition, both arms were held naturally alongside the body. For the arms extended forward condition, participants maintained approximately 90° of shoulder flexion, with both arms parallel to the floor, elbows extended, and palms facing downward. The two postures are illustrated in Figure 1.

Each participant completed six trials representing all combinations of the two arm positions and three test durations (15, 30, and 60 s). The order of the six protocols was determined by drawing lots. Specifically, six identical opaque envelopes, each containing one protocol label, were prepared, and participants drew the envelopes sequentially before testing. A rest interval of at least 1 min was provided between trials and was extended if the participant reported fatigue. Participants were instructed to maintain the prescribed posture and refrain from speaking during data acquisition. A trial was considered invalid if the participant opened their eyes, moved either foot, changed the prescribed arm position, spoke, took a compensatory step, or required physical assistance from the researcher. In such cases, the trial was repeated after sufficient recovery. To ensure safety, a researcher remained beside the participant throughout testing but avoided physical contact unless intervention was required to prevent a fall. Identical testing procedures were used for CSM patients and asymptomatic controls. All records were coded and de-identified before analysis.

#### 2.2.2 Balance assessment parameters

**Figure 2:**
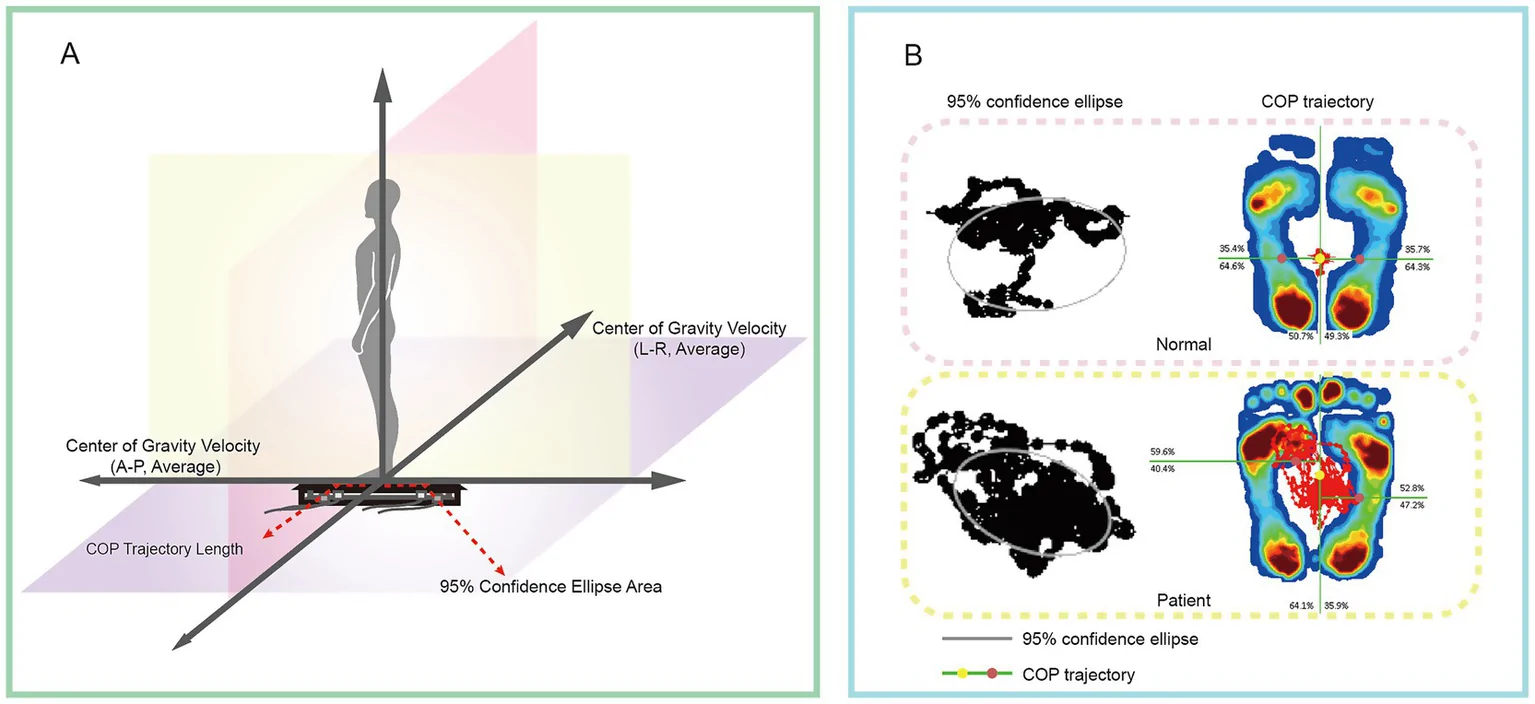
Balance parameters and representative force-platform outputs. **(A)** Schematic illustration of the four balance parameters. **(B)** Representative COP trajectory and 95% confidence ellipse area from a asymptomatic control (top) and a patient with CSM (bottom).

1. Center of pressure (COP) trajectory length (cm): It is the entire distance traveled by the real movement of the center of pressure (COP) of the body in the region of the contact between the foot sole surface and the force platform. It is a direct measure of the degree of activity involved in controlling balance; in other words, the more extensive the path, the greater the number of center of gravity adjustments required to maintain balance and hence the person has relatively low balance stability.
2. 95% confidence ellipse area (cm^2^): It is the region occupied by an ellipse which is placed at the average location of the COP movement throughout the experiment and encloses 95 percent of the COP movement data. Greater area indicates greater variation in the COP action on the support surface indicating less balance stability.
3. Anterior–posterior center of gravity velocity (cm/s): Mean velocity at which COP moves in the front to back (longitudinal) plane of the body during the test. This is an indicator of the effectiveness of the dynamic movements of the COP with regard to the longitudinal axis. The quick alteration of the anterior posterior direction of COP indicates the poor quality of balance and the necessity of rapid changes in the position of COP.
4. Left–right center of gravity velocity (cm/s): It is the average speed of COP movement with regard to the body left to right (lateral) in the course of the test. A high COG speed during this direction indicates poor balance abilities (left to right). Figure 2 will give you more detailed balance assessment reports.

#### 2.2.3 Statistical methods

**Figure 3:**
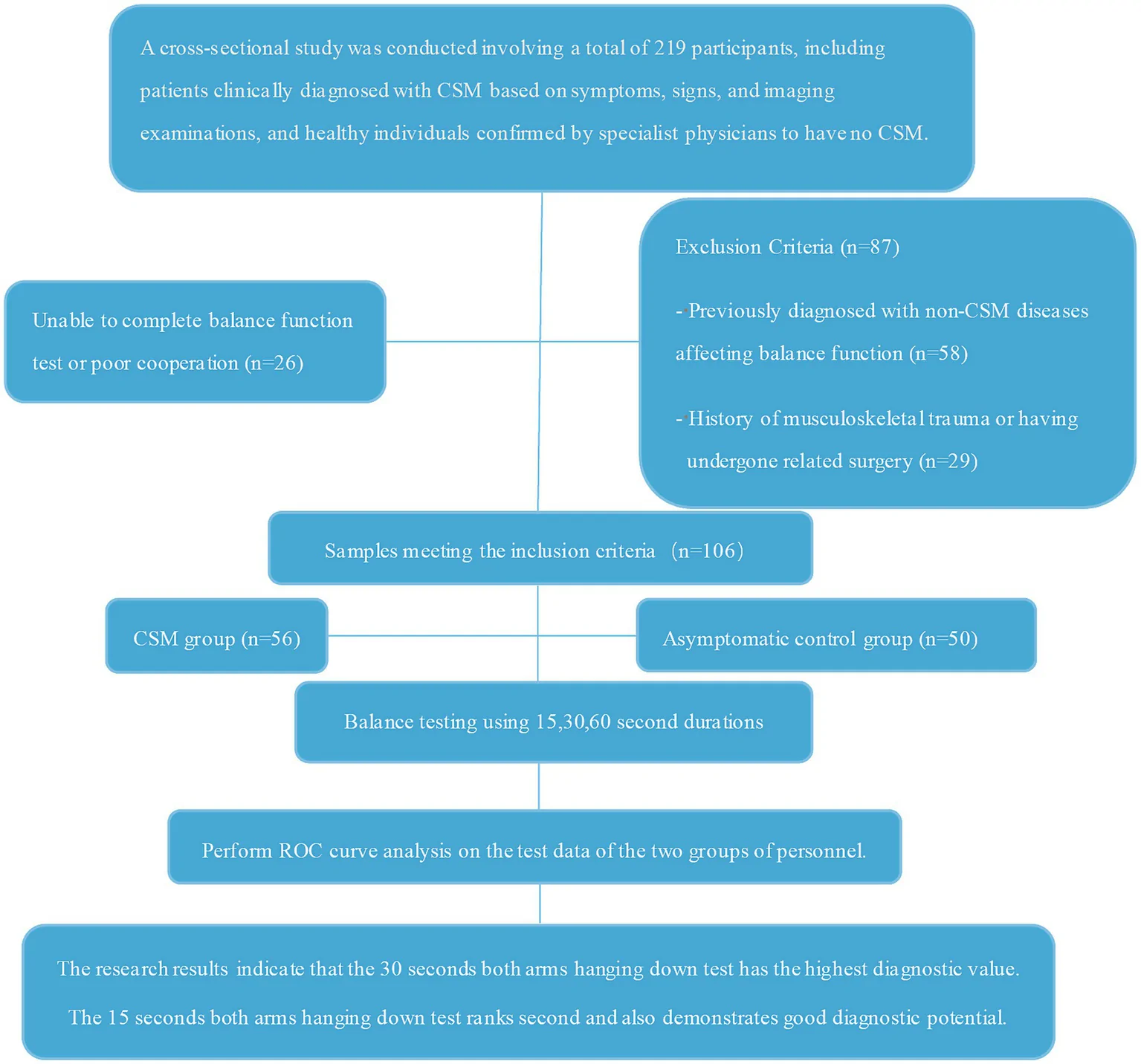
Flowchart of the inclusion and analysis process for balance function samples in CSM patients and healthy controls.

IBM SPSS Statistics for Windows, version 27.0 was used for the statistical analysis. Normally distributed measurement data are expressed as the mean ± standard deviation and are compared with a *t*-test; non-normally distributed data are expressed as the median and interquartile range, and a Mann–Whitney U test is used for comparison. Categorical data are expressed as frequencies and percentages, and the chi-squared test or Fisher’s exact test is used for comparison. ROC curve analysis was performed to determine the diagnostic efficiency of all test methods for CSM in patients at different times. Both sides have *p* < 0.05 and are thus considered statistically significant. Figure 3 shows the experimental flow chart.

## 3 Results

### 3.1 Baseline characteristics of the study participants

The present investigation has enrolled all the 115 CSM patients and 104 asymptomatic controls. Based on the preset exclusion criteria, 59 people were excluded from the CSM group: 25 had other disorders affecting balance function, 20 had a history of musculoskeletal injury or surgery, and 14 could not complete the balance test satisfactorily. A total of 54 people were excluded from the control group: 33 had disorders affecting balance function, 12 were uncooperative, and 9 had a history of musculoskeletal injury or surgery.

After screening, 56 CSM patients and 50 asymptomatic controls were included in the final analysis. The mean age of the CSM group and the control group was 52.4 ± 10.7 years and 54.9 ± 10.7 years; the mean body mass index of the two groups was 25.2 ± 3.0 kg/m^2^ and 25.6 ± 3.8 kg/m^2^. There were 19 smokers (33.9%) in the CSM group and 18 smokers (36.0%) in the control group, and 14 participants (25.0%) and 11 participants (22.0%) reported alcohol consumption. In relation to a history of hypertension, 12 cases (21.4%) in the CSM group were found to be hypertensive; 10 cases (20.0%) asymptomatic control group were diagnosed with hypertension. In terms of a history of diabetes, 7 cases (12.5%) in the CSM group; 6 (12.0%) cases in the asymptomatic control group had been diagnosed. Sex, age, body mass index, smoking, alcohol use, hypertension, and diabetes were not significantly different between these two groups. The basic information of the two groups is shown in Table 1.

**Table 1:** Baseline characteristics of patients with CSM and asymptomatic controls.

Characteristic	CSM Group (*n* = 56)	Asymptomatic control group (*n* = 50)	*p* value
Sex, *n* (%)			0.238
Male	36 (64.3)	26 (52.0)	
Female	20 (35.7)	24 (48.0)	
Age (years), mean ± SD	52.4 ± 10.7	54.9 ± 10.7	0.239
Body mass index (kg/m^2^), mean ± SD	25.2 ± 3.0	25.6 ± 3.8	0.589
Smoking status, *n* (%)	19 (33.9)	18 (36.0)	0.841
Alcohol consumption, *n* (%)	14 (25.0)	11 (22.0)	0.820
Hypertension, *n* (%)	12 (21.4)	10 (20.0)	0.895
Diabetes mellitus, *n* (%)	7 (12.5)	6 (12.0)	0.947

The raw measured values of all balance parameters in the two groups under the different testing methods are summarized in Table 2, which reports the original measurement data rather than area under the curve values. Regardless of testing posture and duration, the COP trajectory length, the 95% confidence ellipse area, the Anterior–posterior center of gravity velocity, and the Left–right center of gravity velocity were all significantly greater in patients with CSM than in healthy controls, with all *p* values below 0.05. It is clear that the type of balance dysfunction present in CSM patients is unique and can be measured properly by using force platform.

**Table 2:** Four balance parameters in patients with CSM and asymptomatic controls across the six testing methods.

Balance parameter	Group	15 s		30s		60s	
		Arms hanging down	Arms extended forward	Arms hanging down	Arms extended forward	Arms hanging down	arms extended forward
95% confidence ellipse area (cm^2^)	Healthy subjects	22.11 ± 14.94	21.91 ± 15.83	29.79 ± 21.13	24.78 ± 15.99	28.19 ± 21.95	30.79 ± 23.38
	CSM patients	47.80 ± 32.97	47.71 ± 36.84	56.53 ± 32.70	49.30 ± 30.15	57.63 ± 34.06	58.39 ± 43.54
Anterior–posterior center of gravity velocity (cm/s)	Healthy subjects	1.687 ± 0.563	1.770 ± 0.568	1.462 ± 0.406	1.564 ± 0.427	1.585 ± 0.621	1.523 ± 0.603
	CSM patients	3.187 ± 2.533	3.431 ± 3.076	3.063 ± 2.544	3.080 ± 2.891	2.900 ± 3.641	3.013 ± 2.416
Left–right center of gravity velocity (cm/s)	Healthy subjects	1.806 ± 0.766	1.767 ± 0.706	1.666 ± 0.707	1.607 ± 0.607	1.566 ± 0.707	1.608 ± 0.606
	CSM patients	3.003 ± 1.576	3.077 ± 1.676	3.076 ± 1.607	2.876 ± 1.608	2.987 ± 1.633	2.796 ± 1.566
COP trajectory length (cm)	Healthy subjects	38.487 ± 8.667	41.263 ± 10.167	72.31 ± 14.59	74.01 ± 17.42	143.32 ± 36.88	150.00 ± 44.83
	CSM patients	70.27 ± 41.86	72.77 ± 49.97	136.26 ± 94.60	133.07 ± 96.30	239.87 ± 189.17	264.07 ± 209.67

*The inter group comparison showed that, in all six conditions (*p* < 0.05), all balance parameter were significantly greater in CSM patients than in healthy controls, which means that the balance function is impaired in CSM patients.

### 3.2 Diagnostic performance of individual balance parameters across different testing methods

**Figure 4:**
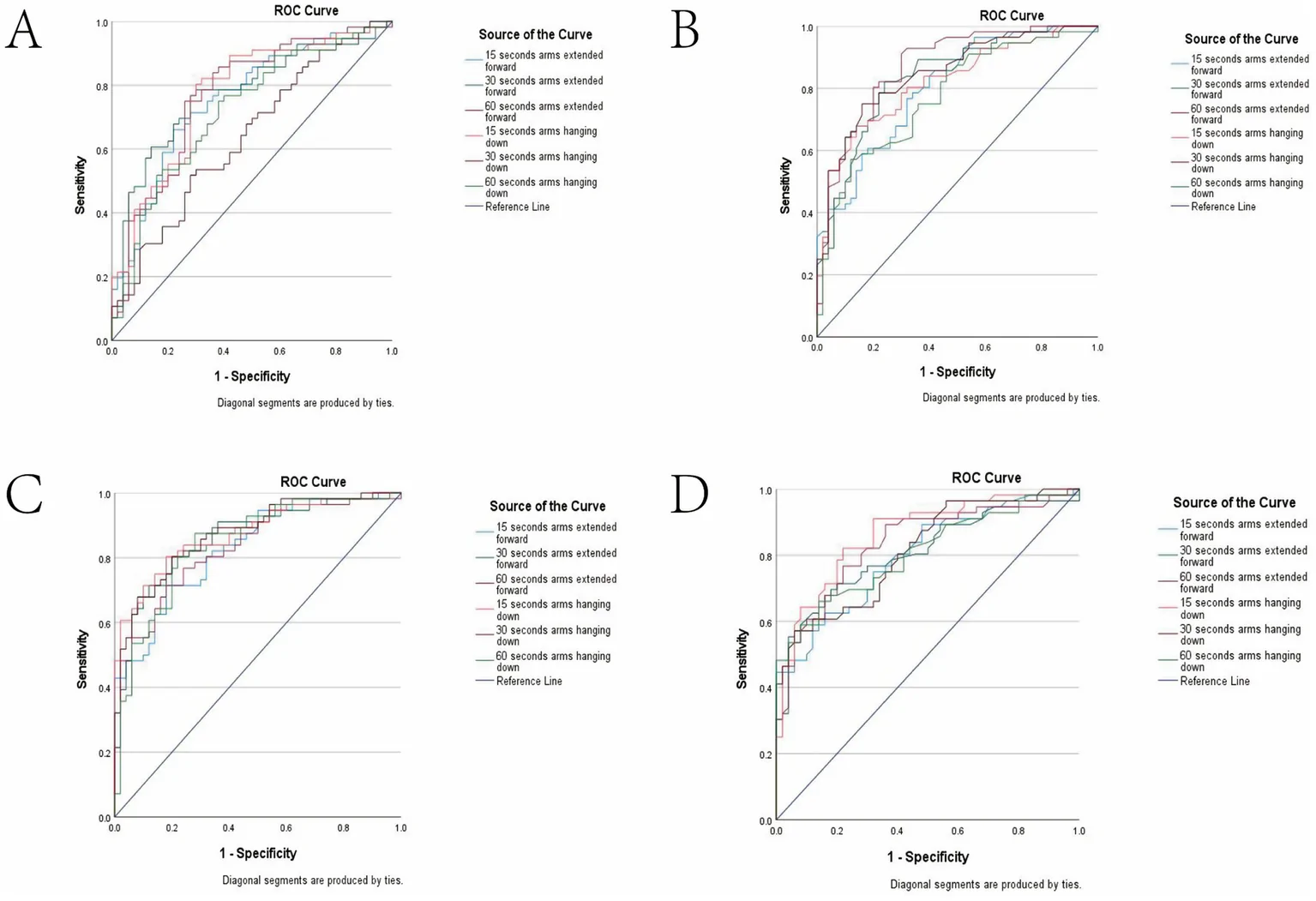
ROC curves for four balance indicators. **(A)** 95% confidence ellipse area (cm²); **(B)** anterior-posterior center of gravity velocity (cm/s); **(C)** center of pressure (COP) trajectory length (cm); and **(D)** left-right center of gravity velocity (cm/s).

In light of the AUC results of the four balance parameters, each balance parameters has a statistically meaningful sensitivity at various combinations of test length and posture, and all the AUC results lie within the range of 0.638 to 0.876. The detailed results are presented in Table 3, and the corresponding ROC curves are shown in Figure 4.

**Table 3:** Observed AUC values for the four individual balance parameters across the six testing methods.

Balance parameter	15 s		30s		60s	
	Arms hanging down	Arms extended forward	Arms hanging down	Arms extended forward	Arms hanging down	Arms extended forward
95% confidence ellipse area (cm^2^) (AUC)	0.772	0.759	0.638	0.773	0.721	0.769
Anterior–posterior center of gravity velocity (cm/s)	0.820	0.804	0.848	0.839	0.773	0.871
Left–right center of gravity velocity (cm/s)	0.863	0.801	0.813	0.807	0.807	0.834
COP trajectory length (cm)	0.876	0.831	0.874	0.861	0.845	0.833

^*^All AUC values were statistically significant (all *p* < 0.05).

### 3.3 Comparative diagnostic efficacy of different testing methods

**Figure 5:**
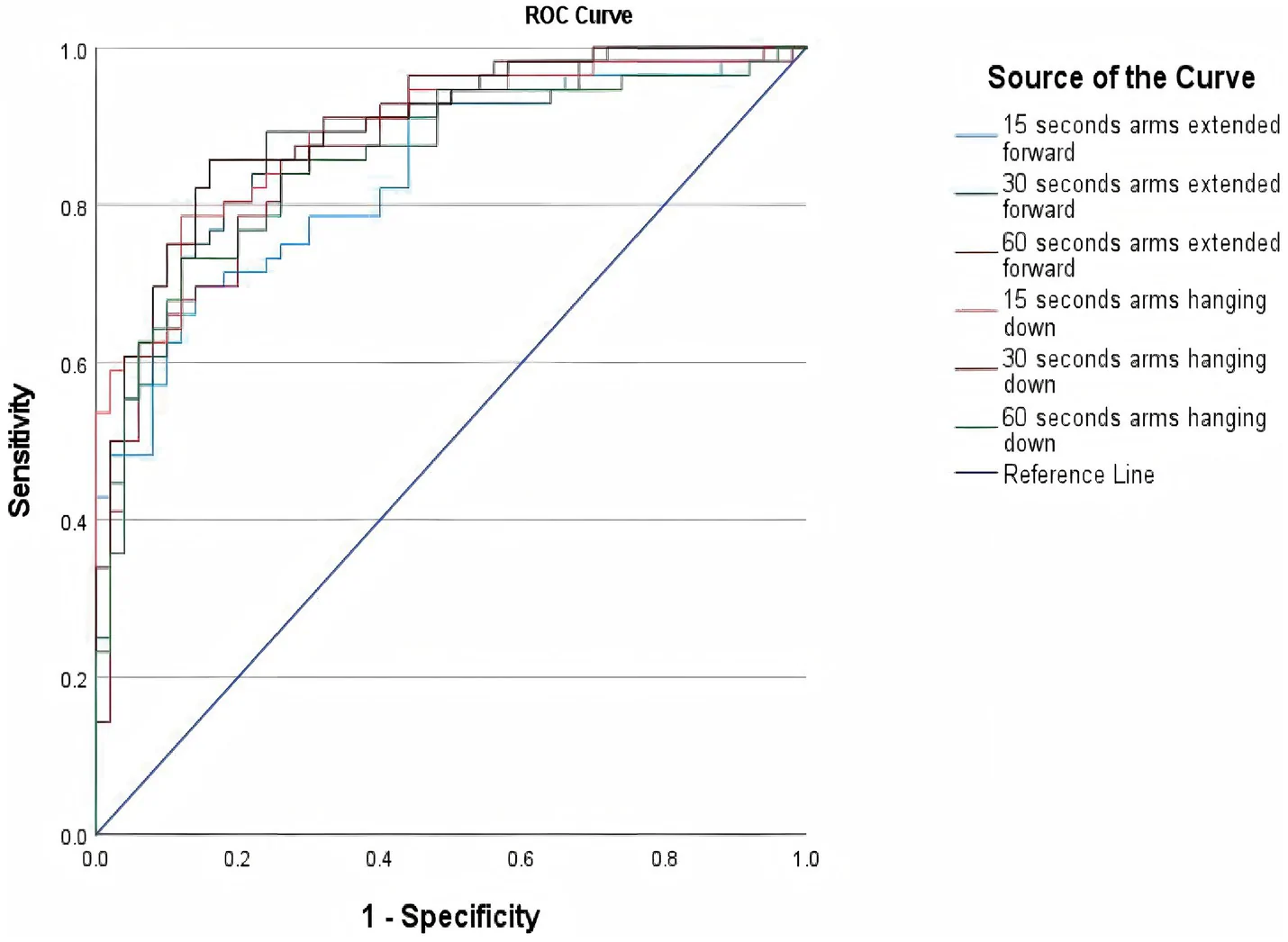
Receiver operating characteristic (ROC) curves for the six testing protocols. The diagonal line represents the reference line.

Each of the six testing methods that were randomly combined with two test postures and three test durations were found to be useful in the classification of CSM patients and asymptomatic controls whose *p*-value did not exceed 0.05. Nevertheless, the discriminative performance differed across testing methods. There is the detailed study of discriminative performance of the balance parameters based on different test postures and time conditions. Table 4 contains the appropriate statistical data and Figure 5 presents the multi indicator composite ROC curve.

**Table 4:** Observed combined AUC values across the six testing methods.

Testing method	Observed AUC	*p* value
15 s arms extended forward	0.840	*p* < 0.05
30 s arms extended forward	0.878	*p* < 0.05
60 s arms extended forward	0.869	*p* < 0.05
15 s arms hanging down	0.890	*p* < 0.05
30 s arms hanging down	0.897	*p* < 0.05
60 s arms hanging down	0.855	*p* < 0.05

The receiver operating characteristic (ROC) curve analysis was one of the tools applied in this research to assess the discriminative performance of each testing protocol for distinguishing patients with CSM from asymptomatic controls. All four balance parameters were integrated into joint variables and combined ROC curves were plotted with six other separate balance testing methods to determine the total discriminative performance of these six testing methods in CSM. The combined ROC analysis showed that the comprehensive diagnostic efficacy of each protocol ranked from highest to lowest as follows: 30s with arms hanging down (AUC = 0.897), 15 s with arms hanging down (AUC = 0.890), 30s with arms extended forward (AUC = 0.878), 60s with arms extended forward (AUC = 0.869), 60s with arms hanging down (AUC = 0.855), and 15 s with arms extended forward (AUC = 0.840).

The results indicate that the 30s arms hanging down test (AUC = 0.897) has the highest diagnostic value. The 15 s arms hanging down test follows closely (AUC = 0.890), also demonstrating good diagnostic potential.

## 4 Discussion

Patients with CSM develop neurological conduction abnormalities caused by spinal cord compression, which directly affect proprioception, motor control, and postural regulation and thereby manifest as balance dysfunction (Fouad et al., 2021; Matz, 2006). Because patients often compensate by adjusting their gait and movement patterns, this dysfunction is frequently inconspicuous and easily missed during routine examination (Mkorombindo et al., 2022). Early and objective identification of balance impairment is clinically important, because prolonged spinal cord compression can lead to irreversible spinal cord injury (Tetreault et al., 2022; Tsukamoto et al., 2020),and detecting balance dysfunction before obvious subjective symptoms appear may help determine the optimal timing of intervention (Tetreault et al., 2022). Force platform testing has been shown to quantify balance impairment in patients with CSM (Mkorombindo et al., 2022). Nevertheless, the testing procedure has not been unified, and the optimal posture and duration remain unclear. The present study therefore evaluated the influence of two standing postures and three testing durations on diagnostic performance, aiming to improve the efficiency of force platform balance testing and to provide a reference for future protocol standardization and outpatient application. Based on the above results, various combinations of standing posture and testing duration are suitable for discriminating between CSM patients and healthy controls. Among the protocols evaluated, the arms hanging down posture had the best overall discrimination ability, and a shorter-duration test in this posture achieved comparable results with higher clinical efficiency; thus, it may be possible to reduce examination time, improve the efficiency of outpatient examination, and decrease the burden on patients.

For both the 15 s and 30s test durations, the arms extended forward position showed lower discriminative performance than the arms hanging down position. Chen et al. (2018a) had found that when a person voluntarily moves their upper limbs, their body position changes as well; thus, the arms help to shift the center of mass during posturing adjustments (Kuks, 2018). The present findings are consistent with this mechanism. Consequently, forward arm posture can decrease the displacement of the center of gravity and increase stability of balance by using such a compensatory mechanism that could possibly impair its ability to recognize small balance defects in patients having CSM. In opposition, during the natural reduction of arms, the extremities of the arms are influenced by gravity and they do not provide additional sources of balance support. Because there is no compensatory mechanism in the upper limbs, this posture is better able to reflect the inability to balance of patients with CSM, indicating greater discriminative ability (Chen et al., 2018b). Interestingly, when the test duration was extended to 60s, the sensitivity in the arms extended forward condition was higher than in the arms hanging down condition; this may be due to muscle fatigue after 1 min of continuous testing. Keeping the arms extended for a longer time increases the strength of the upper-body muscles; healthy people can probably maintain this position better than sick people, and thus the difference between the two groups will be larger. In short, extending the arms forward is not required; a hanging arm position will suffice for the regular assessment. The 30s test performed better than both the 15 s and the 60s test in all postures, but if high efficiency for outpatient use is required, the 15 s test can be selected. In the future, more samples will be taken to explore the actual advantages of the 15 s and 30s lengths for outpatient screening and assessment further.

The Sensitivity of the individual balance parameters varied by test protocol. In the present study, the 60s arms extended forward protocol was more sensitive for the anteroposterior center of gravity velocity, and the 15 s arms hanging down protocol performed best for the mediolateral center of gravity velocity. Ver et al. (2020) found that extending the arms forward increases the anterior–posterior displacement. In addition, our study also found that the effect of arm forward extension on the amplitude of displacement in the anterior posterior direction gradually increased with the extension of test duration, and the discriminative performance of the arms extended forward condition for anterior–posterior center of gravity velocity reached the highest level in the 60s arms extended forward condition. Based on this, future research will focus on the mechanism of the influence of the arms extended forward position on the velocity of the center of gravity in the cand left right directions.

This study has several limitations. Firstly, the number of samples was small and all the participants were admitted to a single healthcare facility, which excluded the population of patients of various regions as well as those whose level of severity is classified. Nevertheless, it can be assumed that the size of the sample in the given study is not sufficient due to clinical heterogeneity of CSM, which limits the generalizability and power of the results. Also, the present paper has not explored the well balanced feature disparity of particular subgroups of CSM patients with enough depth. The next step towards future studies will be the collaboration between multiple centers, the enlargement of the sample and division of the samples by clinical characteristics of CSM and further exploration of the optimal methods to assess the balance of each subgroup in order to come up with a more personalized assessment plan.

## 5 Conclusion

The combination of arm position and testing duration influenced the discriminative performance of force-platform balance assessment in CSM patients. Among the six testing methods, the 30s arms hanging down condition yielded the highest overall AUC, this testing approach has the potential to improve the diagnostic efficiency of CSM in outpatient settings. Individual balance parameters achieved their highest AUC values under different posture–duration combinations, indicating that protocol selection may affect the diagnostic contribution of specific postural variables. These findings provide preliminary evidence for the development of a standardized, efficient, and objective force platform protocol for the assessment of balance impairment in CSM patients.

## Data Availability

The raw data supporting the conclusions of this article will be made available by the authors, without undue reservation.
